# Construction and validation of nomogram prediction model for clinical efficacy of high-dose continuous renal replacement therapy in sepsis patients based on inflammatory response and microcirculation

**DOI:** 10.3389/fmed.2025.1565378

**Published:** 2025-11-27

**Authors:** Chenghua Huang, Zhenfei Huang, Wenfeng Ye, Pingping Liu, Qinglan Li

**Affiliations:** 1 Department of Nephrology, Ganzhou Hospital-Nanfang Hospital, Southern Medical University (Ganzhou People’s Hospital), Ganzhou, China; 2 Department of Critical Care Medicine, Ganzhou Hospital-Nanfang Hospital, Southern Medical University (Ganzhou People’s Hospital), Ganzhou, China; 3 Department of Infectious Diseases, Ganzhou Hospital-Nanfang Hospital, Southern Medical University (Ganzhou People’s Hospital), Ganzhou, China

**Keywords:** sepsis, continuous renal replacement therapy, inflammatory reactions, microcirculation, nomogram prediction model

## Abstract

**Objective:**

To construct a nomogram model for predicting the clinical efficacy of high-dose continuous renal replacement therapy (CRRT) in sepsis patients based on inflammatory response and microcirculation, and to explore its clinical application value.

**Methods:**

A total of 162 sepsis patients who received high-dose CRRT in our hospital were randomly divided into a training set (*n* = 113) and a validation set (*n* = 49) according to a 7:3 ratio. In the training set, multivariate logistic regression was used to analyze the risk factors affecting the treatment effect to construct the nomogram prediction model. The predictive efficacy of the model was assessed using receiver operating characteristic (ROC) curve and calibration curve, which were verified in the validation set. Meanwhile, the decision curve analysis (DCA) was used to evaluate its clinical application value.

**Results:**

The percentages of patients with poor efficacy in the training set and the validation set were 29.20% (33 cases) and 26.53% (13 cases), respectively. Multivariate logistic analysis of the training set showed that high Acute Physiology and Chronic Health Evaluation II (APACHE-II) score, high white blood cell (WBC), procalcitonin (PCT), C-reactive protein (CRP), tumor necrosis factor-alpha (TNF-α) and interleukin-6 (IL-6) levels, and high Sequential Organ Failure Assessment (SOFA) score were the independent risk factors for poor therapeutic effects (all *p* < 0.05). The nomogram model was constructed based on the above results. The model had good calibration and fit in the training set and validation set, with the *C*-index index of 0.865 and 0.836, respectively, the average absolute error of the calibration curve of 0.137 and 0.149, and the *p*-value of Hosmer–Lemeshow test of 0.406 and 0.099, respectively. The area under the ROC curve (AUC) was 0.864 and 0.836 in the training and validation sets, respectively, and the sensitivities and specificities were 0.929, 0.788, and 0.800 and 0.607, respectively.

**Conclusion:**

The nomogram prediction model based on the inflammatory response and microcirculation can effectively predict the clinical efficacy of high-dose CRRT in the treatment of sepsis in patients at an early stage.

## 1 Introduction

Sepsis was a systemic inflammatory response syndrome induced by infection, and its morbidity and mortality remain high, posing a serious threat to human health. Continuous renal replacement therapy (CRRT) has become an important treatment for sepsis patients with acute kidney injury, and high-dose CRRT was expected to improve the prognosis of patients by eliminating inflammatory mediators and other mechanisms (1). However, not all patients with sepsis receiving high-dose CRRT have ideal clinical efficacy. How to accurately predict the patient’s response to treatment and then optimize the treatment strategy has become a key problem to be solved in clinical practice (2). Inflammatory response and microcirculation dysfunction played a vital role in the occurrence and development of sepsis (3). Inflammatory indicators such as white blood cell (WBC) count, procalcitonin (PCT), C-reactive protein (CRP), interleukin-6 (IL-6), and tumor necrosis factor-alpha (TNF-α) can reflect the severity of inflammation. Sublingual microcirculation assessment and other methods can visually present the changes of microcirculation. Relevant parameters such as microvessel density (MVD), perfusion vessel ratio (PPV), and total vascular density (TVD) were of great significance for understanding the condition (4). The establishment of predictive models based on these indicators facilitates early identification of patients who may benefit from high-dose CRRT treatment. The nomogram prediction model constructed based on inflammatory response indicators and microcirculation indicators is expected to provide clinicians with an intuitive, convenient and effective tool for predicting the clinical efficacy of high-dose CRRT in the treatment of sepsis patients, assisting clinical decision-making and improving patient prognosis (5). The purpose of this study was to explore the relationship between these indicators and clinical efficacy, construct and verify the corresponding nomogram prediction model, and provide new ideas and methods for the clinical treatment of sepsis.

## 2 Materials and methods

### 2.1 Subjects

This study was a single-center, retrospective observational study conducted at Ganzhou People’s Hospital. We collected clinical data of 162 sepsis patients who received high-dose CRRT from February 2023 to February 2024. They were randomly divided into a training set (*n* = 113) and a validation set (*n* = 49) according to a 7:3 ratio. The study protocol was approved by the Ethics Committee of Ganzhou People’s Hospital, and informed consent was obtained from all patients or their legal representatives. The research process strictly followed the Declaration of Helsinki. Inclusion criteria: (1) The patient was diagnosed with sepsis according to the internationally recognized diagnostic criteria of sepsis-3 (6); (2) Receiving high-dose CRRT treatment; (3) Age between 18 and 80 years old; (4) Complete clinical data were available. Exclusion criteria: (1) Age <18 years old; (2) End-stage renal disease exists; (3) Combined with malignant tumor end stage; (4) Treatment compliance was poor, and relevant examinations and treatments cannot be completed.

### 2.2 Methods of treatment

All patients received high-dose CRRT treatment, which was defined as a replacement fluid volume of 35–45 mL/(kg·h) according to the Clinical Practice Guidelines for Sepsis Related Acute Renal Injury. Continuous venous venous blood filtration mode treatment was performed using a blood purifier (DX-10, Jianfan Biotechnology Group Co., Ltd.). The blood flow was set at 100–180 mL/min for continuous treatment for 48 h. After 24 to 36 h, the next continuous renal replacement therapy was continued, twice a week. During the treatment, parameters such as the clearance rate and ultrafiltration volume of the blood purifier were adjusted according to the actual condition of the patient.

### 2.3 Data collection

General data of the patients were collected comprehensively, including age, gender, smoking history, drinking history, previous medical history (hypertension, diabetes, cardiovascular disease, chronic kidney disease, etc.), Acute Physiology and Chronic Health Evaluation System II (APACHE II) score, time of using vasoactive drugs, time of using antibiotics, ICU admission time, Sequential Organ Failure Assessment (SOFA) score, and infection site. Meanwhile, the occurrence of complications such as hemorrhage and aggravation of infection during CRRT treatment was recorded in detail. The 28-day survival of the patients was observed, and the 28-day survival was defined as having good clinical efficacy, and the 28-day death was defined as having poor clinical efficacy.

Peripheral venous blood was collected before treatment and inflammatory indicators such as WBC, PCT level, CRP level, IL-6 and TNF-α were detected. PCT was detected using an electrochemical luminescence immunoassay, CRP was detected using an immunoturbidimetric assay, IL-6 and TNF-α were detected using an enzyme-linked immunosorbent assay (ELISA), and WBC count was detected by analyzing blood samples with an automatic blood cell analyzer to obtain the number of white cells.

Sidestream dark-field imaging device (Xuzhou Lihua Electronics Technology Development Co., Ltd.) was used to evaluate the sublingual microcirculation before treatment. The specific measurement process was as follows: (1) Patients were placed in a supine position, and the sublingual mucosa was cleaned with normal saline to remove saliva and food residues. (2) The probe of the device was gently placed 2 cm away from the tip of the tongue, avoiding pressure on the mucosa to prevent microvascular deformation. (3) Three different positions of the sublingual mucosa were imaged, with 20 s of continuous recording per position. (4) Images were analyzed using the device’s dedicated software: MVD was defined as the number of microvessels per square millimeter; PPV was defined as the percentage of perfused microvessels (showing continuous blood flow) among all visible microvessels; TVD was defined as the total length of microvessels per square millimeter. Evaluations were conducted by two independent researchers blinded to patient outcomes; intraclass correlation coefficient (ICC) was used to assess inter-observer agreement (ICC >0.85, indicating good consistency). During the evaluation, parameters such as MVD, PPV, and TVD were recorded.

### 2.4 Statistical analysis

SPSS 26.0 and R 4.2.1 software were used for statistical analysis. If the measurement data conforms to the normal distribution, it was expressed by the mean standard deviation (S), and the comparison between the two groups adopts independent sample *t*-test; if it does not conform to the normal distribution, it was expressed by median (interquartile interval) [M (Q1, Q3)], and Mann–Whitney *U* test was used for comparison between groups. Counting data were expressed by frequency and percentage, and *χ*^2^ test was used for comparison between groups. Univariate analysis was used to screen possible influencing factors, and indicators with *p* < 0.05 were included in the multivariate logistic regression analysis to screen independent influencing factors, and variance inflation factors (VIF) were calculated to exclude multicollinearity (VIF threshold <10). A nomogram model was constructed based on the independent influencing factors. The receiver operating characteristic curve (ROC) was used to evaluate the predictive efficacy of the model, and the area under the curve (AUC) and 95% confidence interval (CI) were calculated. The calibration curve and Hosmer–Lemeshow test were used to evaluate the consistency between the predicted values and the actual values. The decision curve analysis (DCA) was used to evaluate its clinical application value. A *p*-value <0.05 was considered statistically significant.

## 3 Results

### 3.1 Comparison of incidence of poor clinical efficacy and clinical characteristics between the training set and the validation set

A total of 162 sepsis patients who received high-dose CRRT were randomly divided into a training set (*n* = 113) and a validation set (*n* = 49). There were 33 cases (29.20%) with poor clinical efficacy in the training set and 13 cases (26.53%) with poor efficacy in the validation set. There were no significant difference in the rate of poor clinical efficacy and clinical features between the training set and the validation set (all *p* > 0.05) (Table 1).

**Table 1:** Comparison of clinical characteristics between training set and validation set.

Indicators		Training set (*n* = 113)	Validation set (*n* = 49)	*χ*^2^/*t*	*p*
Age (years)		54.33 ± 11.25	53.89 ± 10.89	0.230	0.817
BMI (kg/m^2^)		23.41 ± 3.12	23.15 ± 3.45	0.471	0.637
Gender	Male	63 (55.75)	26 (53.06)	0.100	0.751
	Female	50 (44.25)	23 (46.94)		
Smoking history	Yes	20 (17.70)	9 (18.37)	0.010	0.918
	No	93 (82.30)	40 (81.63)		
Drinking history	Yes	18 (15.93)	8 (16.33)	0.004	0.949
	No	95 (84.07)	41 (83.67)		
History of diabetes	Yes	15 (13.27)	7 (14.29)	0.029	0.862
	No	98 (86.73)	42 (85.71)		
History of hypertension	Yes	34 (30.09)	13 (26.53)	0.210	0.646
	No	79 (69.91)	36 (73.47)		
History of cardiovascular disease	Yes	22 (19.47)	10 (20.41)	0.019	0.890
	No	91 (80.53)	39 (79.59)		
Infected site	Lung	50 (44.25)	22 (44.90)	0.005	0.938
	Abdomen	35 (30.97)	15 (30.61)	0.002	0.963
	Other	28 (24.78)	12 (24.49)	0.001	0.969
Apache II score		18.26 ± 3.87	18.35 ± 4.10	0.133	0.893
Time of using vasoactive drugs (D)		4.13 ± 1.13	4.20 ± 1.01	0.373	0.709
Antibiotic use time (d)		21.36 ± 5.06	20.77 ± 4.87	0.689	0.491
ICU check-in time		19.37 ± 4.65	18.89 ± 5.35	0.576	0.565
SOFA score		7.88 ± 1.98	7.74 ± 2.11	0.405	0.685
WBC (×10^9^/L)		12.35 ± 3.57	12.11 ± 3.87	0.383	0.702
PCT (μg/L)		8.54 ± 4.23	8.32 ± 4.52	0.297	0.766
CRP (ng/L)		2.03 ± 0.48	1.97 ± 0.41	0.762	0.447
IL-6 (ng/mL)		80.64 ± 6.75	81.16 ± 6.77	0.450	0.653
TNF-α (ng/mL)		26.43 ± 3.31	26.45 ± 3.24	0.035	0.971
MVD (mm/mm^2^)		14.23 ± 2.87	14.33 ± 2.79	0.205	0.837
PPV (%)		37.78 ± 2.75	38.03 ± 2.48	0.547	0.585
TVD (mm/mm^2^)		6.34 ± 4.03	6.29 ± 4.16	0.071	0.942

### 3.2 Analysis of risk factors for clinical efficacy of CRRT in sepsis patients in training set

The results of univariate analysis showed that there were statistically significant differences in APACHE II score, WBC, PCT, CRP, TNF-α, IL-6, and SOFA score between patients with good efficacy and patients with poor efficacy (all *p* < 0.05) (Table 2). The clinical efficacy was used as the dependent variable (0 = good, 1 = poor) and the factor *p* < 0.05 in the univariate analysis was used as the covariate for further multivariate logistic regression analysis. The results showed that APACHE II, WBC, PCT, CRP, TNF-α, IL-6, and SOFA scores were the independent risk factors for the 28-day survival of patients with sepsis after high-dose CRRT (all *p* < 0.05) (Table 3). In the regression model, the actual collinearity indicators were as follows: tolerance values of APACHE II score, SOFA score, WBC, PCT, CRP, IL-6, and TNF-α were 0.623, 0.587, 0.712, 0.654, 0.598, 0.631, and 0.605, respectively, (all >0.1). VIF values were 1.605, 1.704, 1.405, 1.529, 1.672, 1.585, and 1.654, respectively, (all <5); condition index was 12.36 (<30); the proportion of variances of multiple covariates with the same eigenvalue was 28.7% (<50%). Hence, there was no collinearity of each covariate.

**Table 2:** Univariate analysis of clinical efficacy of sepsis patients after CRRT treatment in training set.

Indicators		Poor clinical efficacy (*n* = 33)	Good clinical efficacy (*n* = 80)	*χ*^2^/*t*	*p*
Age (years)		55.12 ± 11.05	53.98 ± 10.92	0.502	0.616
BMI (kg/m^2^)		23.05 ± 3.21	23.13 ± 3.08	0.124	0.901
Gender	Male	18 (54.55)	45 (56.25)	0.093	0.759
	Female	15 (45.45)	35 (43.75)		
Smoking history	Yes	6 (18.18)	14 (17.50)	0.007	0.931
	No	27 (81.82)	66 (82.50)		
Drinking history	Yes	5 (15.15)	13 (16.25)	0.021	0.884
	No	28 (84.85)	67 (83.75)		
History of diabetes	Yes	5 (15.15)	10 (12.50)	0.005	0.942
	No	28 (84.85)	70 (87.50)		
History of hypertension	Yes	10 (30.30)	24 (30.00)	0.001	0.974
	No	23 (69.70)	56 (70.00)		
History of cardiovascular disease	Yes	7 (21.21)	15 (18.75)	0.090	0.763
	No	26 (78.79)	65 (81.25)		
Infected site	Lung	14 (42.42)	36 (45.00)	0.062	0.802
	Abdomen	10 (30.30)	25 (31.25)	0.075	0.783
	Other	9 (27.27)	19 (23.75)	0.155	0.693
Apache II score		20.22 ± 4.13	17.88 ± 3.44	3.096	0.002
Time of using vasoactive drugs (D)		4.20 ± 1.22	3.92 ± 1.08	1.206	0.230
Antibiotic use time (d)		21.25 ± 5.21	19.97 ± 4.87	1.244	0.215
ICU check-in time		19.63 ± 4.81	19.20 ± 4.53	0.450	0.653
SOFA score		8.56 ± 2.24	7.52 ± 1.78	2.612	0.010
WBC (×10^9^/L)		14.43 ± 4.33	12.48 ± 3.20	2.645	0.009
PCT (μg/L)		10.03 ± 4.36	7.88 ± 3.82	2.609	0.010
CRP (ng/L)		2.21 ± 0.52	1.92 ± 0.49	2.810	0.005
IL-6 (ng/mL)		83.62 ± 7.46	79.97 ± 6.92	2.491	0.014
TNF-α (ng/mL)		28.36 ± 3.24	26.64 ± 3.16	2.611	0.010
MVD (mm/mm^2^)		13.32 ± 2.51	14.02 ± 2.98	1.186	0.238
PPV (%)		37.52 ± 2.63	37.95 ± 2.74	0.767	0.444
TVD (mm/mm^2^)		6.41 ± 4.26	5.38 ± 4.03	1.215	0.227

**Table 3:** Multivariate logistic regression analysis of clinical efficacy after CRRT in sepsis patients.

Indicators	*β*	Standard error	Wald	*p*	OR	95% CI
APACHEII score	0.183	0.064	8.212	0.004	1.201	1.060–1.362
SOFA score	0.283	0.112	6.354	0.012	1.328	1.065–1.655
WBC	0.156	0.062	6.321	0.012	1.169	1.035–1.320
PCT	0.131	0.053	6.212	0.013	1.140	1.028–1.264
CRP	1.187	0.441	7.224	0.007	3.276	1.379–7.782
IL-6	0.076	0.032	5.577	0.018	1.079	1.013–1.149
TNF-α	0.169	0.068	6.211	0.013	1.184	1.037–1.352

### 3.3 Development of nomogram prediction model for clinical efficacy of patients with sepsis after CRRT treatment

**Figure 1:**
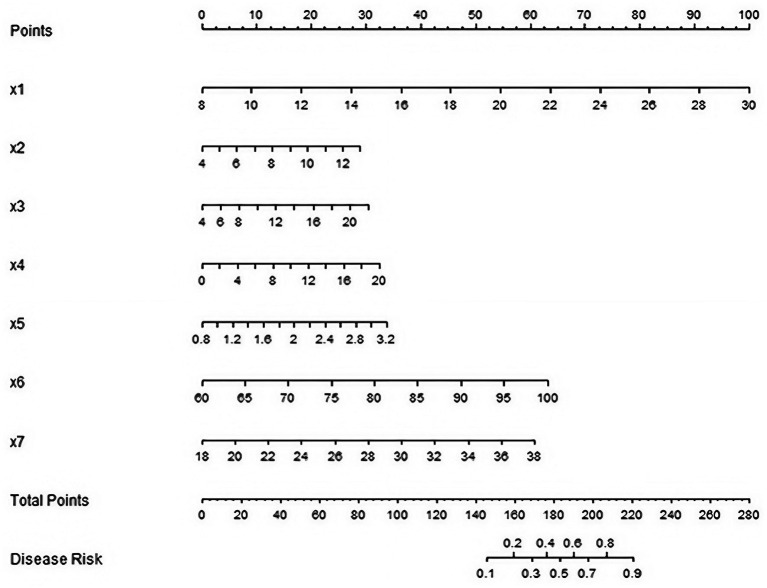
Nomogram prediction model for clinical efficacy of patients with sepsis after CRRT treatment (X1: APACHE II score, X2: SOFA score, X3: WBC, X4: PCT, X5: CRP, X6: IL-6, X7: TNF-α).

A nomogram prediction model for clinical efficacy of sepsis patients after CRRT treatment was constructed based on the independent risk factors identified by multivariate logistic regression analysis. Each factor was corresponding to the corresponding scale. The scores were found on the scale according to the actual values of various factors in patients and added to obtain the total score. The 28-day survival prediction probability was read out from the probability scale on the right side of the nomogram by the total score (Figure 1).

### 3.4 Assessment and validation of nomogram prediction model for clinical efficacy of CRRT in sepsis patients

**Figure 2:**
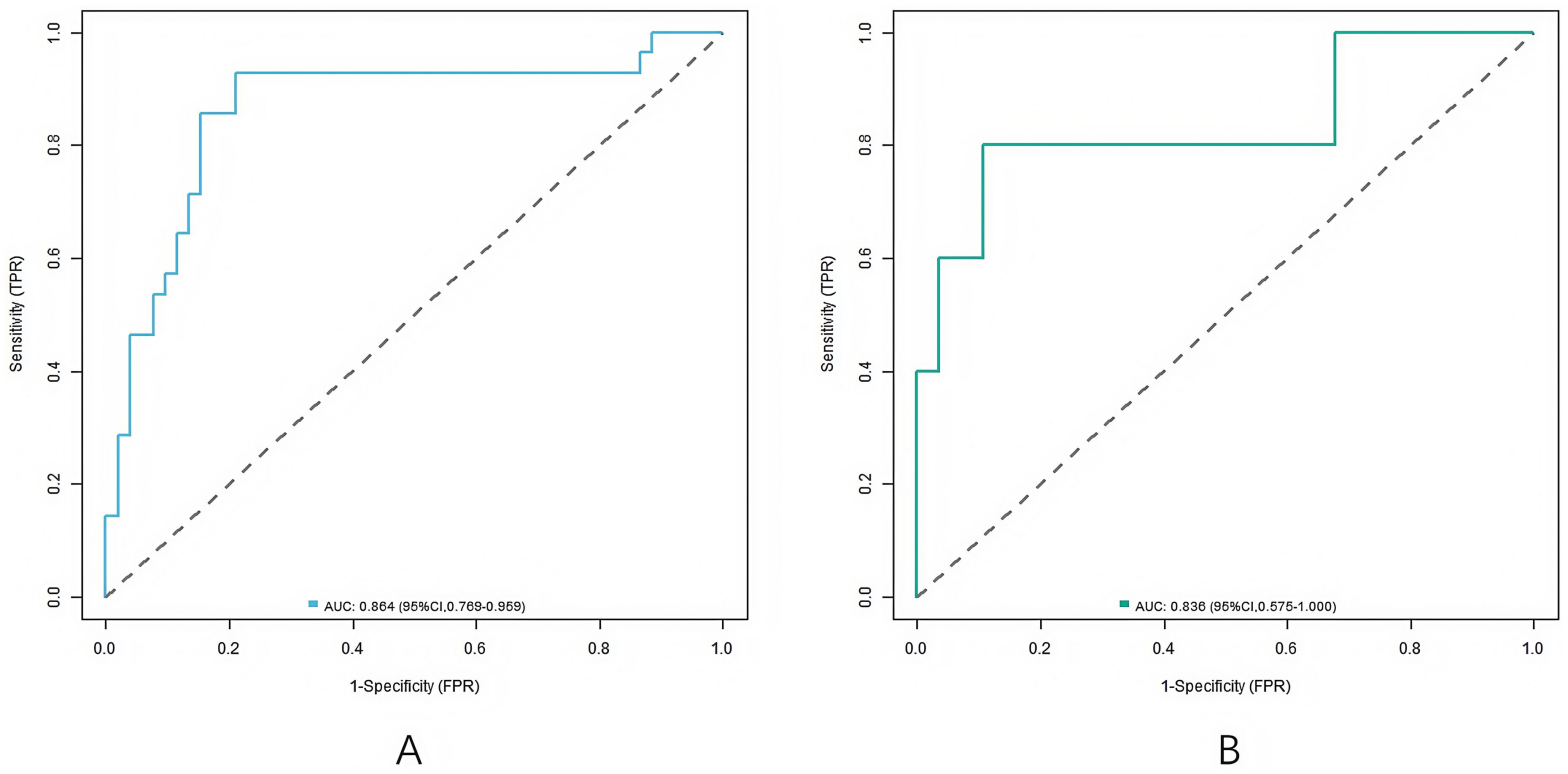
Receiver operating characteristic curves (**A**: the training set, **B**: the validation set).

**Figure 3:**
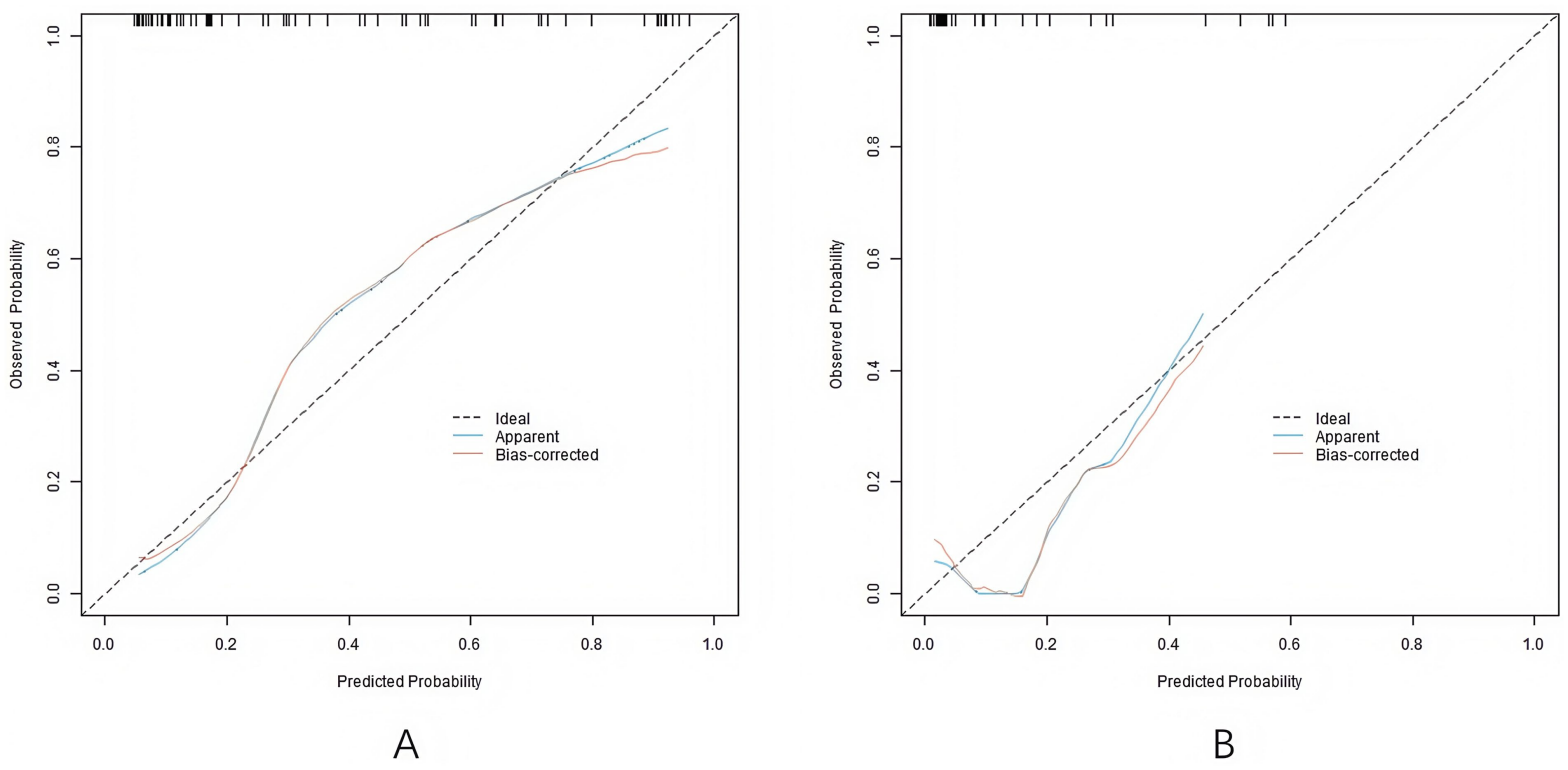
Calibration curves (**A**: the training set, **B**: the validation set).

In the training set and validation set, the *C*-index values of the nomogram models were 0.865 and 0.836, respectively, the mean absolute errors of the calibration curves for the predicted values and the true values were 0.137 and 0.149, respectively, and the Hosmer–Lemeshow test results were *χ*^2^ = 8.283, *p* = 0.406 and *χ*^2^ = 13.393, *p* = 0.099, respectively (Figure 2). The ROC curves were shown in the training set and the validation set. The AUC of the nomogram model for predicting the clinical efficacy of CRRT in sepsis patients after CRRT treatment was 0.864 (95% CI: 0.769–0.959) and 0.836 (95% CI: 0.575–1.000), respectively. The sensitivity and specificity were 0.929, 0.788 and 0.800, 0.607, respectively (Figure 3).

### 3.5 Analysis of decision curve analysis of nomogram prediction model for clinical efficacy of CRRT in sepsis patients

**Figure 4:**
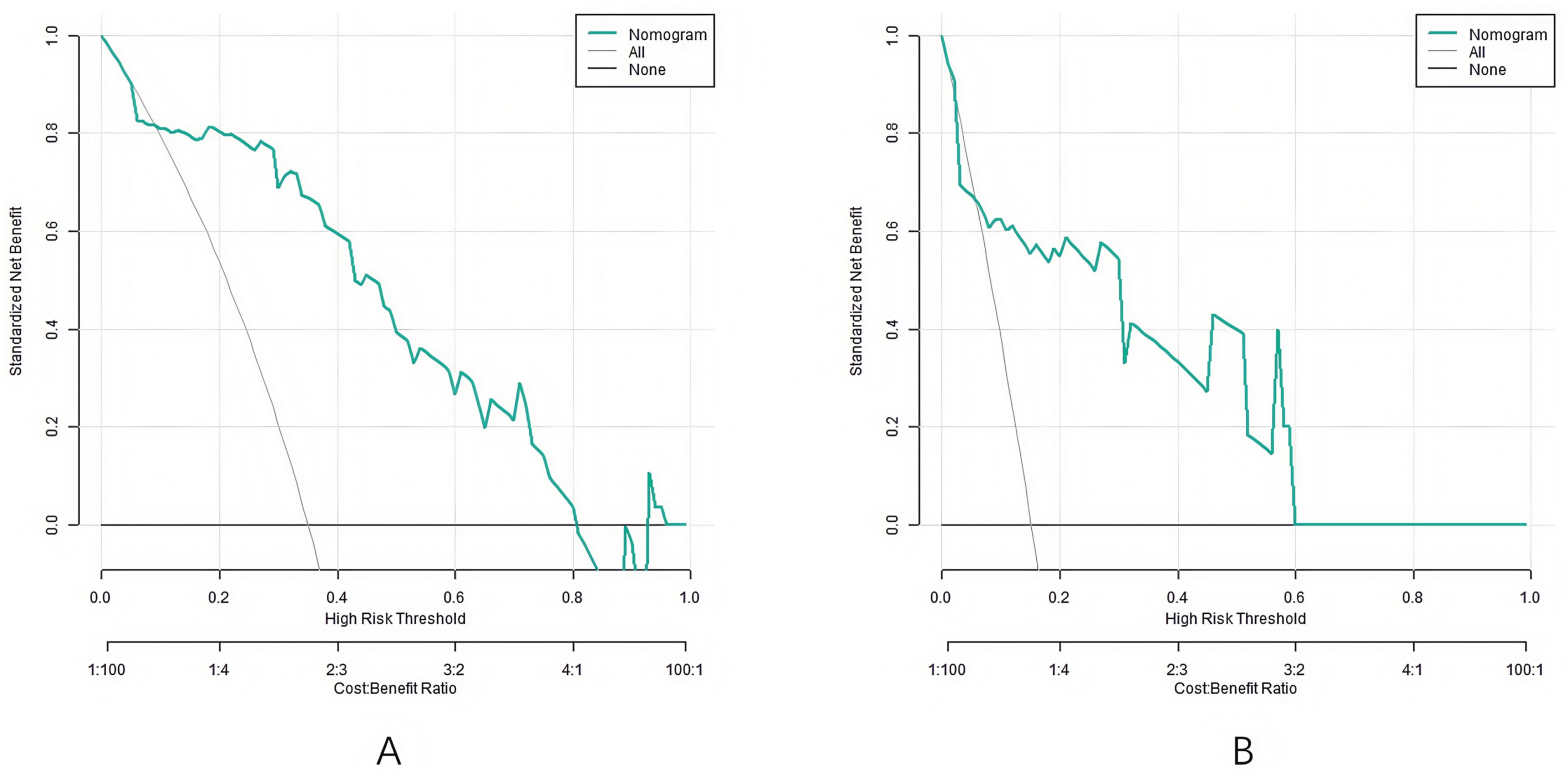
Decision curves (**A**: the training set, **B**: the validation set).

The decision curve analysis showed that when the threshold was within the range of 0.10–0.90, the application of Nomogram column line model to predict the clinical efficacy of CRRT in sepsis patients has more clinical benefits than the decisions that were considered as good or bad (Figure 4).

## 4 Discussion

In this study, a total of 162 sepsis patients receiving high-dose CRRT were enrolled and randomized into a training set (*n* = 113) and a validation set (*n* = 49). The 28-day mortality rates, reflecting poor treatment efficacy, were 29.20 and 26.53%, respectively. Multivariate logistic regression analysis identified several independent risk factors significantly associated with poor clinical efficacy, including elevated APACHE II scores, high white blood cell counts, increased levels of PCT, CRP, TNF-α, and IL-6, as well as higher SOFA scores. Third, the nomogram prediction model demonstrated strong discriminative ability, with *C*-index values of 0.865 in the training set and 0.836 in the validation set, along with satisfactory calibration. Sepsis, as a severe systemic inflammatory response syndrome, has a high morbidity and mortality, which brings great challenges to clinical treatment. High-dose CRRT has been widely used in the treatment of sepsis. However, there were significant differences in the response of different patients to CRRT. Accurate prediction of the therapeutic effect was essential to optimize the treatment strategy and improve the prognosis of patients. In this study, we constructed a nomogram prediction model for the clinical efficacy of high-dose CRRT in the treatment of sepsis based on the inflammatory response and microcirculation indicators, providing an intuitive and practical prediction tool for clinicians.

The APACHE II score combines multiple factors including the patient’s acute physiological state, age, and chronic health status to provide a comprehensive assessment of the severity of the patient’s condition. In this study, logistic analysis showed that APACHE II score was an independent risk factor for poor efficacy of high-dose CRRT in the treatment of sepsis in patients. A high APACHE II score generally means that a patient was in a critical condition at the time of admission, may have multiple organ dysfunction, and was poorly compensated by the body. This makes patients receiving high-dose CRRT less tolerant to treatment and hard to endure various stress reactions that may occur in the treatment process (7). For example, patients with high scores may have difficulties in maintaining effective blood circulation and gas exchange during CRRT treatment due to cardiopulmonary dysfunction, which may affect the treatment outcome. In addition, patients with severe diseases may have more severe inflammatory reactions and microcirculation disorders. Even if receiving high-dose CRRT treatment, it was difficult to quickly correct the internal environment disorder, leading to poor prognosis (8). Leukocytes were an important part of the immune system, and changes in their number and function can reflect the immune state and the degree of inflammatory response. In this study, we found that high WBC level was an independent risk factor for poor therapeutic effect. In sepsis, the immune system was activated, and white blood cells proliferate and release a variety of inflammatory mediators to resist the invasion of pathogens (9). However, excessive inflammatory response can lead to waterfall release of inflammatory mediators, triggering a systemic inflammatory response syndrome that can cause damage to the body. A high level of WBC suggests that the inflammatory response of the body was relatively severe, and bacterial infection may be difficult to control. Even if high-dose CRRT treatment was performed, it was difficult to effectively remove excessive inflammatory mediators, thereby affecting the therapeutic effect (10). In addition, leukocyte adhesion and aggregation may occur during inflammation, resulting in microcirculation disorder, further aggravating tissue ischemia and hypoxia and affecting the recovery of organ function (11). PCT is a biomarker associated with bacterial infection that is often significantly elevated in patients with sepsis. Our results suggest that high PCT levels were associated with poor outcomes with high-dose CRRT. An increase in PCT levels reflects the severity of the bacterial infection and the activity of the inflammatory response. A high level of PCT suggests that the infection may be serious and the inflammatory response persists. High-dose CRRT can clear some inflammatory mediators, but it was difficult to completely inhibit the strong inflammatory response (12). In addition, PCT may be involved in the pathophysiological process of sepsis through a variety of pathways, such as inducing inflammatory cells to release more inflammatory mediators and damaging vascular endothelial cells, thereby affecting the therapeutic effect and patient prognosis (13). CRP is a classic acute phase-reactive protein that rises rapidly within 6–12 h of the onset of inflammation, making it a sensitive marker for monitoring the dynamics of the inflammatory response in sepsis. Notably, high CRP levels emerged as a highly significant independent risk factor for poor efficacy of high-dose CRRT (OR = 3.276, *p* = 0.007) in our study. This OR value indicated that every unit increase in CRP (ng/L), the risk of poor clinical efficacy (28-day mortality) increases by 2.276 times, highlighting its strong predictive weight in the model—even higher than some other inflammatory indicators. The underlying mechanism linking high CRP to poor CRRT efficacy can be explained in three key ways. Uncontrolled systemic inflammation: Persistently elevated CRP in sepsis patients was a direct sign of an unresolved systemic inflammatory response. Although high-dose CRRT can remove small-molecule inflammatory mediators (e.g., TNF-α, IL-6) through hemofiltration, CRP itself (a 118-kDa protein) was not efficiently cleared by the DX-10 blood purifier used in this study. As a result, high CRP levels persist, continuing to activate inflammatory cells (e.g., neutrophils, macrophages) and trigger the cascade release of pro-inflammatory cytokines. This sustained inflammation overwhelms the body’s compensatory capacity, leading to progressive organ dysfunction (e.g., renal, respiratory) that cannot be reversed by CRRT alone. Aggravated microcirculation disorder: CRP has a high affinity for damaged vascular endothelial cells. It binds to endothelial cell surface receptors (e.g., FcγRIIb) to promote leukocyte adhesion and aggregation, forming microthrombi in capillaries. This directly reduces microvessel perfusion (reflected by lower PPV in our microcirculation assessment) and exacerbates tissue ischemia-hypoxia—especially in vital organs like the kidneys and lungs. Since CRRT relies on adequate tissue perfusion to deliver its therapeutic effects (e.g., removing metabolic wastes), impaired microcirculation directly diminishes the efficacy of CRRT. CRP also modulates the tissue repair process by inhibiting the proliferation and migration of endothelial progenitor cells. In sepsis patients, high CRP levels delay the repair of damaged vascular and organ tissues, prolonging the duration of organ dysfunction. This means that even if CRRT stabilizes the patient’s hemodynamics, the slow tissue recovery caused by high CRP still leads to poor long-term outcomes. Our findings were further supported by previous studies (14, 15). TNF-α and IL-6, important pro-inflammatory cytokines, play a key role in the inflammatory cascade of sepsis. In this study, we found that high levels of TNF-α and IL-6 were both independent risk factors for poor therapeutic effects. TNF-α can activate inflammatory cells, leading to a large release of inflammatory mediators, causing tissue damage and organ dysfunction. IL-6 can promote the activation and proliferation of inflammatory cells, regulate the immune response, and affect the synthesis of acute phase proteins in the liver to further aggravate the inflammatory response. In sepsis patients, the increased TNF-α and IL-6 levels indicate severe inflammatory response. High-dose CRRT treatment may be difficult to rapidly reduce the levels of these cytokines, resulting in the persistent inflammatory response, which affects the treatment effect and patient prognosis (16, 17). SOFA scoring system was used to assess the degree of organ dysfunction in patients with sepsis, covering multiple systems including respiratory, coagulation, liver, cardiovascular, central nervous system and kidney. Our results indicate that a high SOFA score was an independent risk factor for poor efficacy in high-dose CRRT. A high SOFA score indicates severe organ dysfunction in patients, and dysfunction of multiple organs will affect the overall function and compensatory ability of the body (18). For example, respiratory dysfunction may lead to hypoxia in the body, affecting cell metabolism and function; renal dysfunction will affect the removal of metabolic wastes in the body and the maintenance of water and electrolyte balance. In this case, even if high-dose CRRT treatment was performed, it was difficult to improve organ function in a short time, thus affecting the therapeutic effect and the survival of the patient (19).

The nomogram demonstrated robust performance in both training and validation cohorts. It exhibited excellent discriminative ability, with *C*-index values of 0.865 and 0.836, respectively. Calibration was satisfactory, with mean absolute errors of 0.137 and 0.149, and non-significant Hosmer–Lemeshow test results (*p* = 0.406 and 0.099). The AUC values further confirmed strong predictive accuracy (0.864 and 0.836). Decision curve analysis indicated the model provided superior clinical net benefit within a practical threshold range, supporting its potential utility in guiding individualized treatment decisions for sepsis patients undergoing CRRT (20). Although some results have been achieved in this study, there were still some limitations. First, this study conducted only internal validation, and no external validation. This was mainly because external validation needs to collect a large amount of case data from other medical institutions, and it faces many difficulties in actual operation. Different medical institutions may have differences in the diagnostic criteria, treatment options and data recording method of sepsis, which will affect the consistency and comparability of data. In addition, obtaining data from other medical institutions requires a large amount of time and energy, involving ethical and legal issues of data sharing, as well as communication and coordination with other hospitals. These factors limit the implementation of external validation. Second, since this study was a single-center study, there may be some limitations in the representation of samples (21). The results of a single-center study may be affected by factors such as the medical level and patient group characteristics of the center, and its popularity may be limited. Future research should actively carry out multi-center, large sample research, and external validation to further verify and improve the nomogram prediction model, improve the accuracy and universality of the model (22). Finally, the disappointing performance of microcirculation parameters (MVD, PPV, TVD) in predicting efficacy may be due to: (1) The small sample size (*n* = 162) leading to insufficient statistical power to detect weak associations; (2) The single time point of microcirculation assessment (only before CRRT), which fails to reflect dynamic changes during treatment; (3) Possible measurement bias (e.g., slight differences in sublingual imaging positions). Future studies may choose dynamic microcirculation indicators (e.g., changes in PPV before and after treatment) or combine other microcirculation markers (e.g., microvascular flow velocity) to improve predictive value.

In summary, this study developed a nomogram model for predicting clinical outcomes of high-dose CRRT in sepsis patients. The model demonstrated robust performance in both training and validation sets, showing potential for supporting individualized treatment strategies. Nonetheless, further multi-center, large-sample studies and external validation are warranted to enhance its generalizability. With continued refinement, this tool may offer valuable insights for sepsis management and contribute to improved patient survival.

## Data Availability

The raw data supporting the conclusions of this article will be made available by the authors from Ganzhou Hospital-Nanfang Hospital, Southern Medical University (Ganzhou People’s Hospital), without undue reservation.
